# Intermittent Complete Atrioventricular Block Secondary to Scrub Typhus in a Patient With Hypothyroidism: A Case Report From Nepal

**DOI:** 10.7759/cureus.88936

**Published:** 2025-07-28

**Authors:** Sajjan Thapa, Rishika Rawal, Prabal Tiwari, Amir R Giri, Sushant Guragain

**Affiliations:** 1 Internal Medicine, Dhulikhel Hospital, Dhulikhel, NPL; 2 Internal Medicine, Kantipur Hospital, Lalitpur, NPL

**Keywords:** bradycardia, complete heart block, holter monitoring, hypothyroidism, scrub typhus

## Abstract

Scrub typhus, a common febrile illness in South Asia, can rarely cause life-threatening myocarditis with complete heart block. We report a 43-year-old man presenting with a one-week history of fever, shortness of breath, and abdominal pain. Laboratory findings showed transaminitis, hypoalbuminemia, elevated C-reactive protein, and mild hypokalemia, suggesting an infectious etiology. Electrocardiography (ECG) and 24-hour Holter confirmed complete atrioventricular (AV) block with P-wave and QRS dissociation. Although temporary pacing was initially considered due to the severity of the block, the patient remained hemodynamically stable, prompting a systematic evaluation for reversible causes. Scrub typhus IgM (enzyme-linked immunosorbent assay) was positive, confirming the diagnosis. Other potential causes such as Lyme disease (low local incidence), ischemic heart disease (ruled out by the absence of chest pain, normal troponin levels, and unremarkable ECG findings), AV-nodal blocking agents (no relevant medication history), and autoimmune or infiltrative conditions (not clinically suspected) were systematically excluded. Although hypokalemia was identified and corrected, it failed to improve the heart block, suggesting it was not the underlying cause. Similarly, hypothyroidism was unlikely to explain the early improvement, as levothyroxine typically takes weeks to take effect. Scrub typhus-associated myocarditis was thus considered the probable cause. The patient was treated with doxycycline 100 mg twice daily and levothyroxine 25 μg daily with continuous cardiac monitoring. Within 72 hours, the fever resolved, and the ECG showed restoration of a sinus rhythm with sinus bradycardia, confirming AV block resolution. Doxycycline was continued for 14 days. This case illustrates that scrub typhus can serve as a reversible etiology for bradyarrhythmias and cardiac conduction abnormalities. While complete heart block frequently necessitates urgent pacing, it is imperative to actively pursue reversible causes. This underscores the significance of clinical vigilance in endemic regions, where early recognition and prompt initiation of antibiotic therapy can avert serious cardiac complications and reduce the necessity for invasive interventions.

## Introduction

Scrub typhus is a rickettsial disease prevalent in many parts of South Asia, including Nepal [1]. It is caused by *Orientia tsutsugamushi*, an obligate intracellular bacterium transmitted through the bite of infected larval mites (chiggers) [2]. Scrub typhus contributes to around 19.3% of acute undifferentiated febrile illnesses in Nepal, as reported by a systematic review [3]. For its diagnosis, IgM enzyme-linked immunosorbent assay (ELISA) kits are most widely used in the country, offering a sensitivity between 79.73% and 87.68% and a specificity ranging from 93.43% to 95.99% [4]. The illness typically presents as an acute febrile syndrome accompanied by chills, malaise, arthralgia, abdominal discomfort, and vomiting [5]. Although most cases are self-limiting, untreated infections can lead to serious multisystem involvement. Complications may include septic shock, acute respiratory distress syndrome, pneumonitis, acute kidney injury, central nervous system manifestations such as meningitis or meningoencephalitis, myocarditis, disseminated intravascular coagulation, upper gastrointestinal hemorrhage, and multiorgan dysfunction syndrome [6].

Cardiovascular involvement is well recognized and may present as myocarditis, congestive heart failure, tachyarrhythmias, or conduction abnormalities such as first-degree atrioventricular (AV) block, largely due to direct endothelial and myocyte invasion. These manifestations usually occur during the acute phase of infection, typically after the onset of febrile symptoms. Most published cardiovascular cases from India and Thailand describe mild conduction delays or atrial arrhythmias, while complete, reversible third-degree AV block resolving within 72 hours of doxycycline treatment remains uncommon [7,8]. We report what is likely the first documented case of scrub typhus-associated complete heart block in Nepal, occurring in a patient with coexisting hypothyroidism. This case highlights the importance of considering scrub typhus as a reversible cause of unexplained bradyarrhythmias and heart block in endemic regions to ensure timely diagnosis and treatment.

## Case presentation

A 43-year-old male presented to the emergency department with a seven-day history of fever, headache, malaise, and dry cough. Then he subsequently developed shortness of breath at rest, right upper quadrant abdominal pain, and bilateral lower limb swelling persisting for four days. The patient did not report orthopnea, chest pain, palpitations, syncope, rash, bleeding, or any changes in mental status. The patient had not traveled recently, had no chronic illnesses, and did not take any medications.

On initial examination, the patient appeared ill and febrile. Vital signs showed an irregular pulse rate of 54 beats per minute (bpm), blood pressure of 110/80 mmHg, respiratory rate of 22 breaths per minute, oxygen saturation of 88% on room air, and a temperature of 39°C. There was evidence of pitting edema in both lower extremities. On abdominal examination, mild tenderness was elicited in the right upper quadrant, without accompanying guarding or rigidity. Cardiovascular and respiratory examinations were normal. No lymphadenopathy or eschar was noted.

The laboratory evaluation showed a total leukocyte count of 9.2×10⁹/L, with 48% neutrophils and 44% lymphocytes, and a normal platelet count. Additionally, mild transaminitis was present, along with hypoalbuminemia and an elevated C-reactive protein (CRP) level. Renal function remained within normal limits. A detailed overview of the hematological, hepatic, and renal parameters is provided in Table 1. Thyroid function tests indicated hypothyroidism, demonstrated by an elevated thyroid-stimulating hormone (TSH) level and reduced free thyroxine (Table 2). 

**Table 1:** Laboratory parameters on admission and after treatment ALP: Alkaline phosphatase; ALT: Alanine aminotransferase; AST: Aspartate aminotransferase; CRP: C-reactive protein; TLC: Total leukocyte count.

Investigation	Results (before)	Results (after)	Unit	Reference range
TLC	9.2	8.2	×10³/µL	4.0–11.0
Neutrophils	48	57	%	45–75
Lymphocytes	44	33	%	20–45
Monocytes	6	7	%	2–8
Eosinophils	2	3	%	1–6
Platelet count	306	383	×10³/µL	150–450
AST	65	56	IU/L	5–40
ALT	56	51	IU/L	5–40
ALP	111	-	U/L	<115 (M), <105 (F)
Total protein	6.3	-	g/dL	6.0–8.3
Albumin	2.8	-	g/dL	3.5–5.0
Urea	24.0	22.0	mg/dL	10–45
Creatinine	1.1	0.8	mg/dL	0.6–1.3
Sodium	141.0	137.0	mEq/L	135–148
Potassium	3.3	4.0	mEq/L	3.5–5.3
CRP	>90	<2	mg/L	<10.0

**Table 2:** Thyroid and metabolic laboratory profile T3: Triiodothyronine; T4: Thyroxine; TSH: Thyroid-stimulating hormone.

Investigation	Results	Unit	Reference range
Free T3	1.25	pg/mL	2.0–4.4
Free T4	0.84	ng/dL	0.9–1.7
TSH	33.51	µIU/mL	0.5–5.0
D-dimer	4.45	mg/L	0–0.5
Calcium (corrected)	8.8	mg/dL	8.4–10.2
Phosphorus	6.3	mg/dL	2.5–4.5
Magnesium	1.9	mg/dL	1.7–2.4

**Figure 1:**
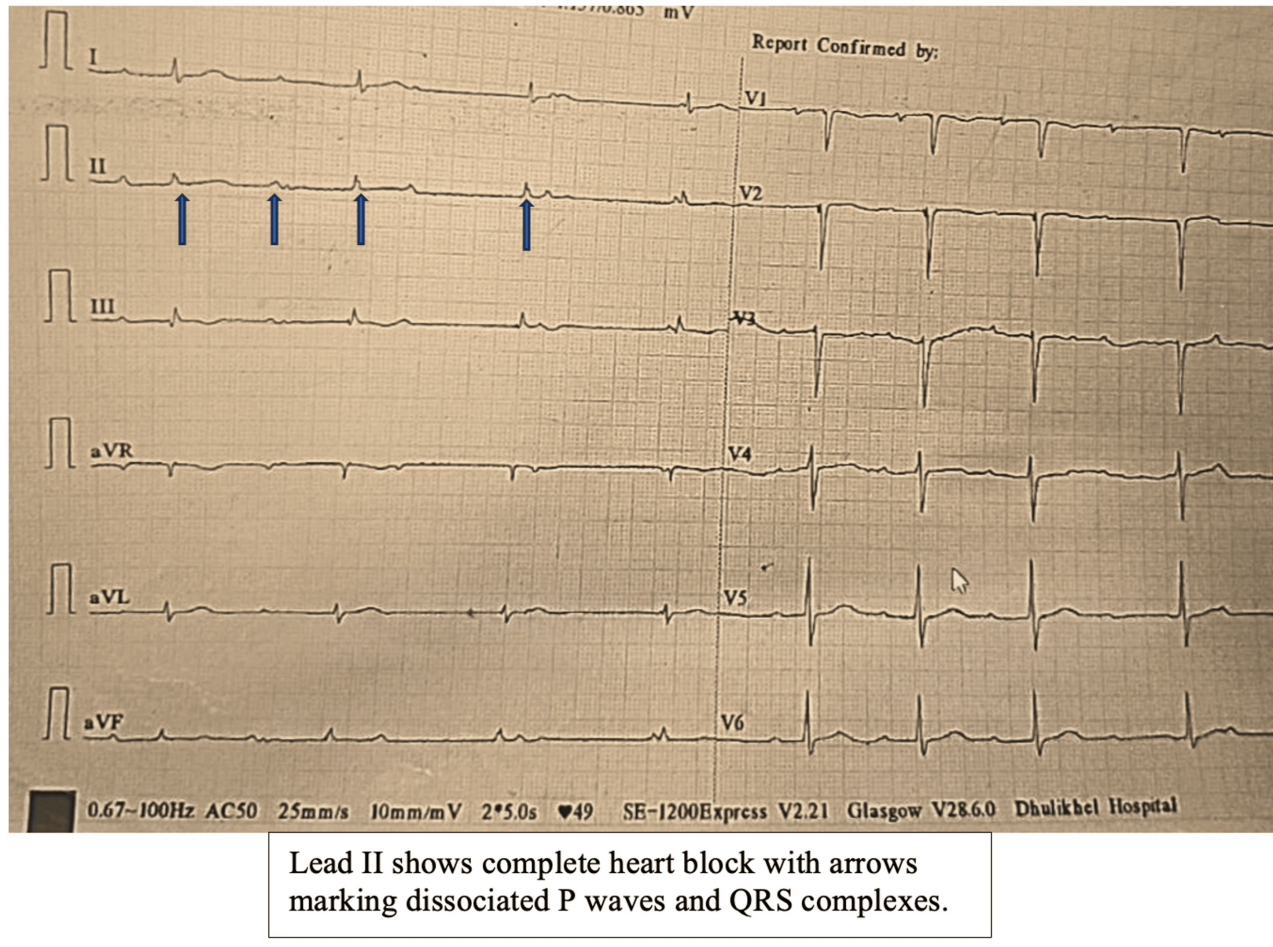
Electrocardiographic evidence (lead II) of complete heart block with independent atrial and ventricular activity

The patient’s initial 12-lead electrocardiography (ECG) demonstrated complete heart block, characterized by complete dissociation between P waves and QRS complexes, bradycardia, and an irregular ventricular rate (Figure 1). Additionally, low-voltage QRS complexes were noted in the limb leads, along with a QS complex in lead V1, raising suspicion for myocardial involvement such as myocarditis or acute ischemic heart disease. However, the absence of chest pain, lack of ST-segment changes, and persistently negative serial troponin I levels effectively ruled out ischemic cardiac disease. Given the persistent AV block, a 24-hour Holter monitor was planned to do continuous heart rhythm monitoring and to further evaluate the case. In light of these findings and the clinical picture, serological testing for infectious causes was conducted. This testing identified a positive scrub typhus IgM (ELISA), while screening for human immunodeficiency virus, hepatitis B surface antigen, hepatitis C virus, and the Venereal Disease Research Laboratory test returned negative results (Table 3).

**Table 3:** Diagnostic serology: positive scrub typhus and negative differentials HBsAg: Hepatitis B surface antigen; HCV: Hepatitis C virus; HIV: Human immunodeficiency virus; NS1: Nonstructural protein 1; VDRL: Venereal Disease Research Laboratory.

Investigations	Result
Scrub typhus IgM	Positive
*Leptospira* IgM	Negative
Dengue (NS1, IgM, and IgG)	Negative
HIV, HBsAg, HCV, and VDRL test	Non-reactive

Arterial blood gas analysis revealed mild hypoxemia (pH: 7.42, pCO₂: 34.6 mmHg, and pO₂ :30 mmHg), prompting initiation of continuous positive airway pressure. Although a temporary pacemaker was considered due to the severity of the heart block, the patient remained hemodynamically stable, allowing for comprehensive evaluation of reversible causes. The patient was admitted to the intensive care unit, with suspected scrub typhus-associated myocarditis and conduction abnormalities. Treatment included intravenous doxycycline (100 mg twice daily), levothyroxine (25 mcg once daily), and supportive care. Hypokalemia detected on laboratory testing was managed with oral potassium chloride. Continuous cardiac monitoring was maintained due to the risk of progression to high-grade AV block.

**Figure 2:**
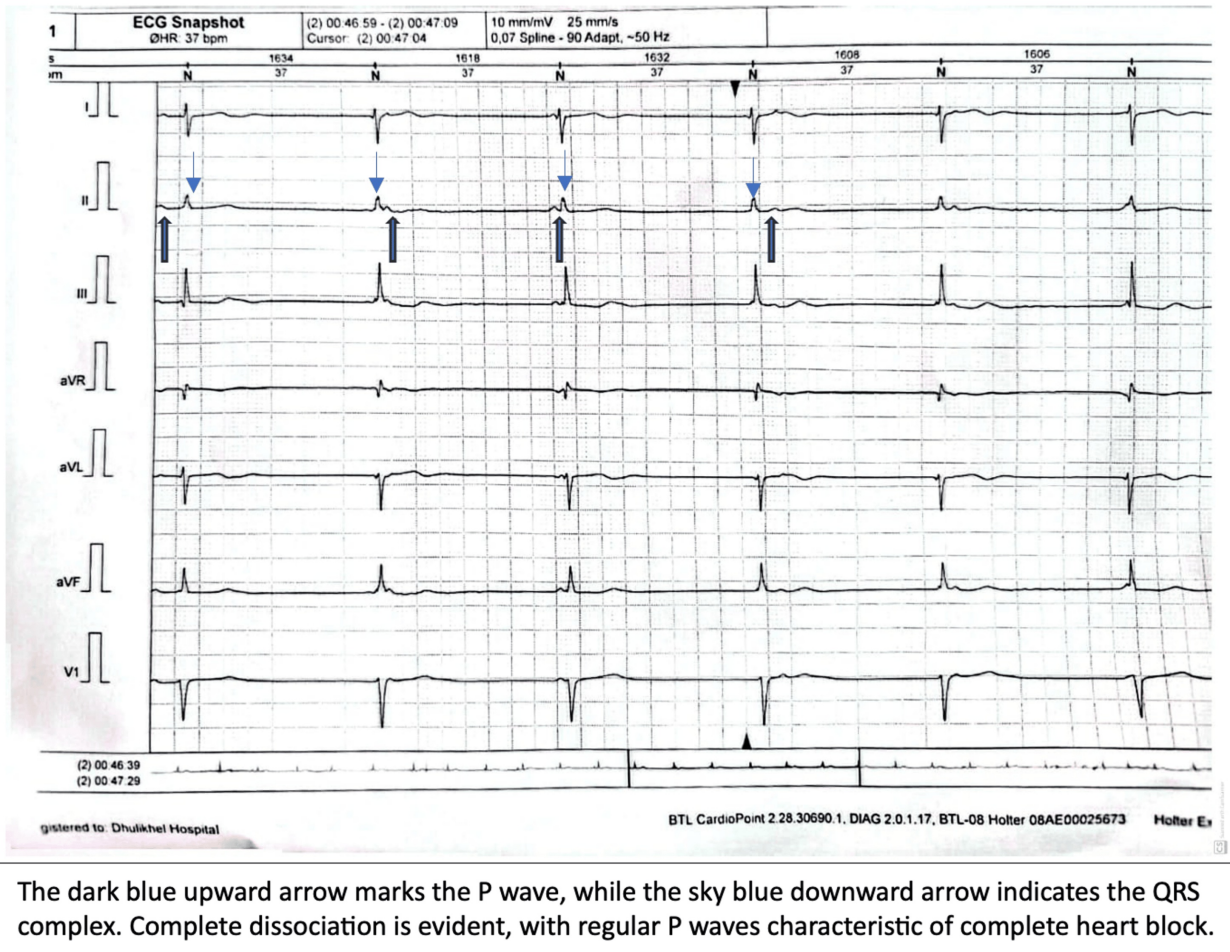
Holter image showing intermittent complete heart block

**Figure 3:**
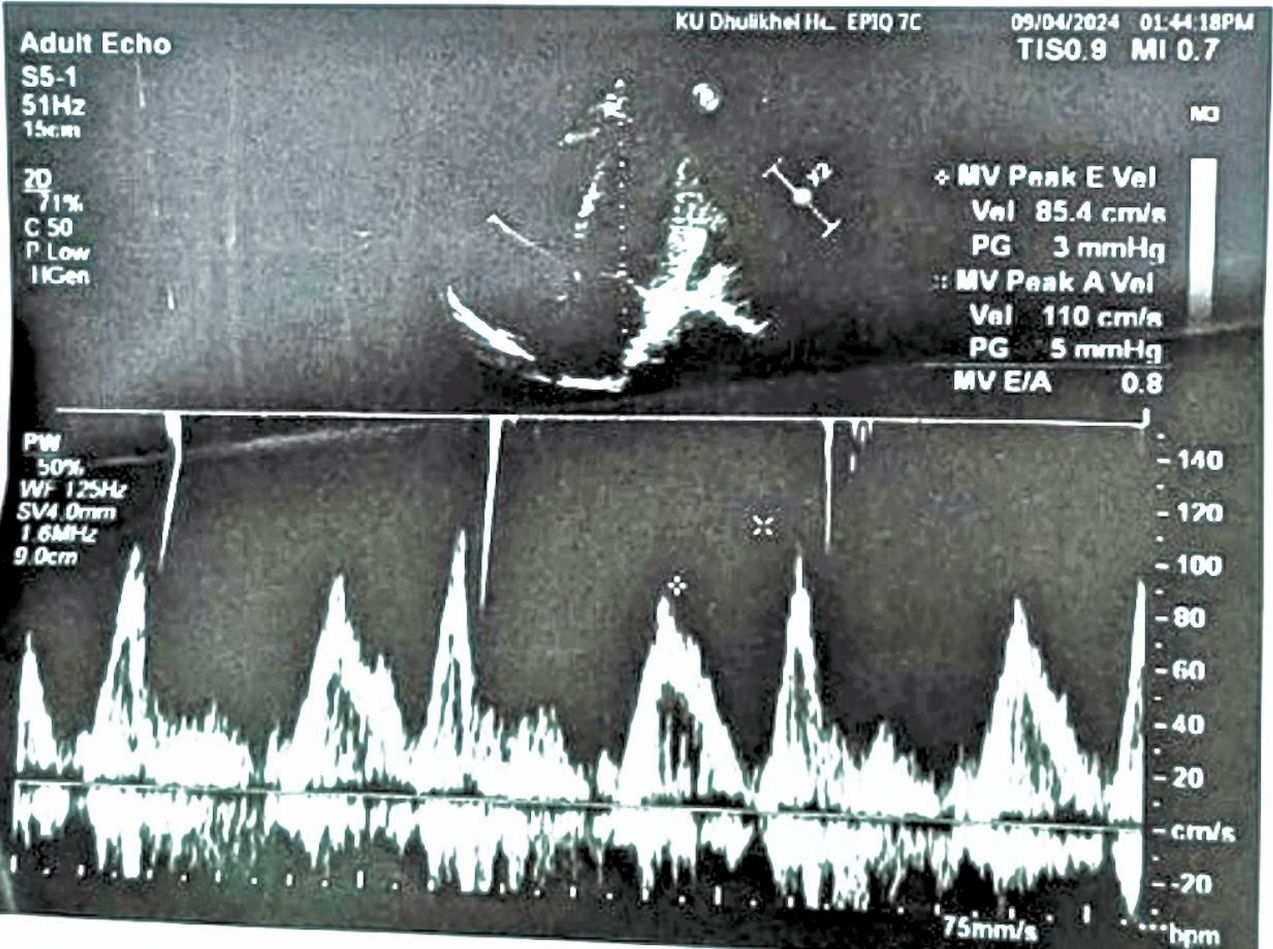
Transthoracic echocardiogram illustrating mitral regurgitation likely secondary to myocarditis

**Figure 4:**
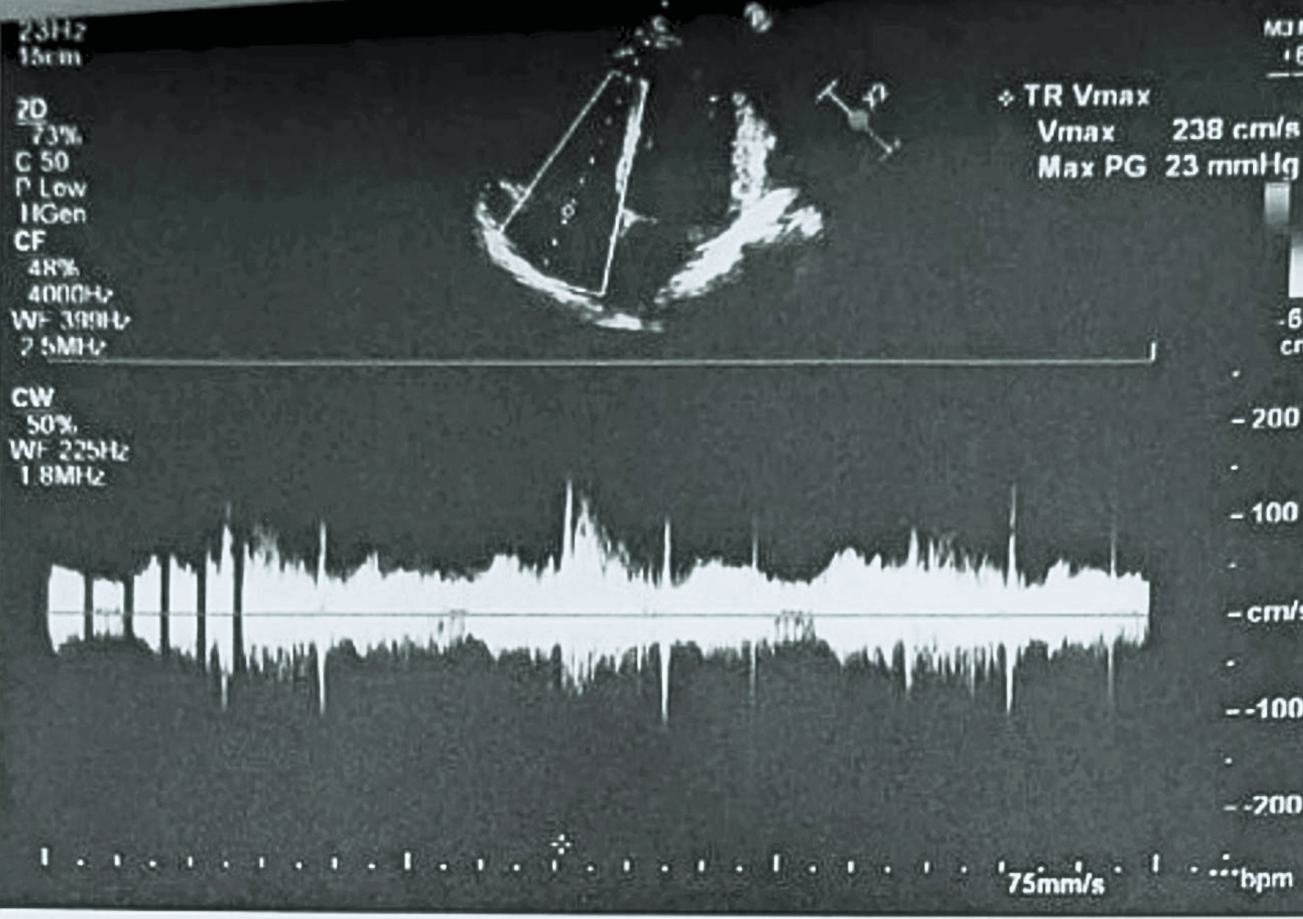
Echocardiography showing systolic regurgitant flow across the tricuspid valve probably due to myocarditis

Over the next 24 hours, the patient’s oxygen requirements increased, accompanied by persistent electrocardiographic abnormalities. Laboratory evaluation revealed a markedly elevated D-dimer level of 4.45 mg/L (reference range: 0-0.5 mg/L). However, chest computed tomography (CT) angiography showed no evidence of pulmonary embolism or other abnormalities. Continuous Holter monitoring identified intermittent complete AV block with a heart rate as low as 27 bpm and pauses lasting up to 6.5 seconds, confirming the initial diagnosis of complete heart block (Figure 2). Transthoracic echocardiography demonstrated mild tricuspid and mitral regurgitation based on qualitative color Doppler assessment, with preserved left ventricular ejection fraction of 55% (Figures 3, 4). Additionally, chest CT revealed mild pleural effusions and cardiomegaly.

**Figure 5:**
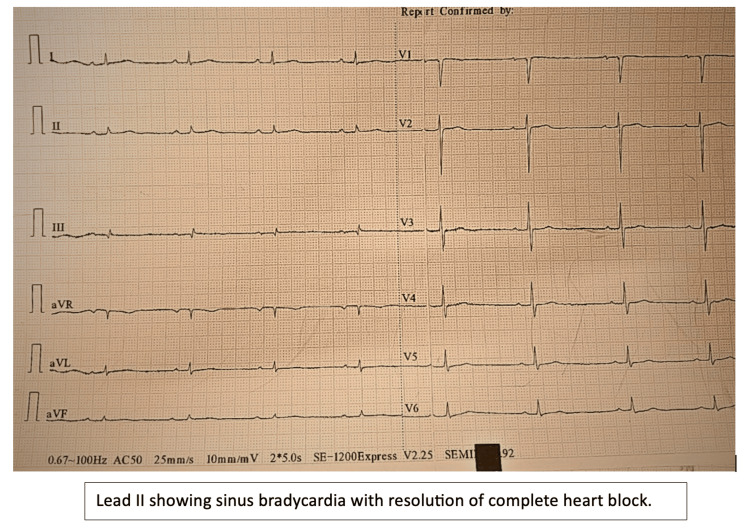
Electrocardiogram demonstrating sinus bradycardia with a regular atrial rhythm and prolonged R-R intervals

Following treatment, the patient demonstrated significant clinical improvement. Oxygen requirements decreased progressively, and they were eventually weaned off. A repeat ECG revealed sinus bradycardia with regular rhythm and, importantly, no evidence of complete heart block (Figure 5). These findings confirmed the reversibility of conduction abnormalities related to scrub typhus myocarditis.

The patient was discharged after normalization of his cardiac conduction abnormalities. He was instructed to complete the prescribed 14-day course of doxycycline and continue thyroid hormone replacement therapy. A follow-up visit was scheduled for six weeks later. Regular cardiology follow-up was recommended to monitor for any potential late cardiac complications, such as persistent conduction disturbances or myocardial dysfunction, allowing for timely intervention if necessary. The patient was also counseled on the importance of adherence to medication and follow-up appointments to ensure optimal recovery and long-term cardiac health.

## Discussion

Scrub typhus, an infection caused by *Orientia tsutsugamushi*, is most commonly seen in the “tsutsugamushi triangle,” a region stretching from Pakistan to Japan and northern Australia, including Nepal [9]. The disease, with an incubation period of 6-21 days, is transmitted through the bite of infected chigger larva, which often leaves an eschar at the site of the bite [6]. Scrub typhus often presents with non-specific symptoms and atypical clinical features, as observed in our case. Given that scrub typhus is a leading cause of undifferentiated febrile illness in endemic regions, early diagnostic consideration is essential [10].

Scrub typhus-induced myocarditis involves immune-mediated damage triggered by the host's inflammatory response, including elevated levels of cytokines such as tumor necrosis factor-alpha, interleukin-6, and interleukin-1β, which can induce cardiomyocyte injury. Vasculitis and endothelial dysfunction compromise myocardial perfusion, contributing to myocardial inflammation. The disease may involve the interventricular septum, coronary arteries, and cardiac valves, causing arrhythmia [11]. Clinical manifestations often include tachycardia, impaired cardiac function, and rhythm disturbances. If diagnosis and treatment are delayed, these complications may progress, significantly increasing the risk of morbidity and mortality [12].

In our patient, markedly elevated CRP (>90 mg/L), hypoalbuminemia, and transaminitis reflected a severe inflammatory response typical of complicated scrub typhus. These biochemical markers have been strongly associated with severe disease and cardiac involvement, including myocarditis [13]. We also ruled out thromboembolic causes of the elevated D-dimer using the Wells score and CT pulmonary angiography. Although non-specific, D-dimer can be elevated due to the systemic inflammatory state observed in scrub typhus.

Various arrhythmias, such as sinus arrhythmia, sinus or relative bradycardia, sinus tachycardia, inverted T waves, U waves, ST-segment elevation, QT prolongation, and heart blocks, have been reported in scrub typhus [11]. Our case demonstrated an intermittent complete heart block, confirmed by Holter monitoring (heart rate 27 bpm, pauses up to 6.5 seconds). Given this significant conduction abnormality, we decided to further analyze and compare the various types of heart blocks reported in association with scrub typhus. A summary of these findings is presented in Table 4 [5,8,14-17].

**Table 4:** Summary of ECG conduction abnormalities in scrub typhus: incidence, reversibility, and references AV: Atrioventricular; ECG: Electrocardiography.

S.N.	ECG changes	Incidence	Type of research	Reversibility	Reference
1	Second-degree AV block	1	Original research	Yes, within 48 hours	Gupta et al. [5]
2	First-degree AV block	6	Original research		Choi et al. [17]
	Third-degree AV block	1	Original research		
	Right bundle branch block	5	Original research		
	Left bundle branch block	1	Original research		
3	First-degree AV block	1	Original research		Thipmontree et al. [8]
	Third-degree AV block	1	Original research		
4	Third-degree AV block	1	Original research	Yes	Dalal et al. [15]
5	Third-degree AV block	1	Original research	Yes, within 48 hours	Yadav et al. [14]
6	Left bundle branch block	3	Original research		Pannu et al. [16]
	Right bundle branch block	2	Original research		

In our patient, the complete heart block observed on Holter monitoring showed the rhythm reverted to first-degree AV block within 72 hours of initiating doxycycline therapy, without the need for temporary pacing. This temporal correlation strongly suggests that the conduction abnormality was reversible and likely related to the underlying inflammatory process induced by scrub typhus. This is consistent with previous studies by Gupta et al. [5], Yadav et al. [14], and Dalal et al. [15], which have reported resolution of high-grade AV blocks within 48 hours of treatment.

The echocardiographic findings in our study predominantly showed preserved ejection fraction, with mild tricuspid regurgitation being the most common structural abnormality. These results align with the observations by Sivasubramanian et al. [11], who reported a significant disparity between ECG and echocardiographic findings in patients with scrub typhus. In their study, 97% of patients demonstrated ECG abnormalities such as sinus tachycardia and conduction blocks, while only 19% exhibited structural changes on echocardiography, including pericardial effusion, tricuspid regurgitation, and myocarditis [11].

Although our patient was also diagnosed with hypothyroidism, the rapid resolution of ECG abnormalities within 72 hours of initiating doxycycline strongly suggests that scrub typhus-induced myocarditis and conduction disturbances were the primary causes of the heart block. Thyroxine replacement therapy typically requires several weeks to exert significant cardiac effects, making it unlikely to have contributed to this early improvement [18]. Nonetheless, treating hypothyroidism remains important for long-term cardiovascular health and the prevention of future complications.

Ruling out other reversible causes of complete heart block was essential before attributing it to an infectious etiology. Ischemic heart disease was excluded by the absence of chest pain, ischemic ECG changes, and negative troponin. Drug‑induced AV block was ruled out, as the patient was not taking AV‑nodal blocking agents, and correction of mild hypokalemia did not improve conduction. Autoimmune or infiltrative conditions such as sarcoidosis and systemic lupus erythematosus were not clinically suspected, given the lack of supportive systemic features. Lyme carditis, another recognized cause of transient complete heart block, was considered highly unlikely due to its extremely low incidence in Nepal. With these reversible causes excluded, the confirmed diagnosis of scrub typhus, coupled with severe systemic inflammation and multiorgan involvement, strongly supports scrub typhus-associated myocarditis as the underlying etiology of the conduction abnormality.

Myocarditis should be suspected in patients presenting with new, unexplained cardiac symptoms, ECG changes, elevated cardiac biomarkers, arrhythmias, or ventricular dysfunction [19]. Our clinical evaluation, biochemical markers, Holter monitoring, and echocardiography provided valuable insights into myocarditis with complete heart block due to scrub typhus, particularly in endemic regions. Although cardiac MRI and endomyocardial biopsy remain the gold standards for definitive diagnosis, their use must be balanced against clinical context, availability, and impact on management. In our patient, scrub typhus was confirmed by IgM ELISA, and the presence of severe systemic inflammation, multi-organ involvement, and conduction abnormalities was consistent with complicated scrub typhus. Rapid clinical and ECG improvement within 72 hours of doxycycline initiation suggested that advanced imaging or biopsy would unlikely have altered treatment.

Endomyocardial biopsy, despite its diagnostic value, is invasive and carries risks such as perforation and arrhythmia, which are particularly concerning in patients with high-grade conduction block. Cardiac MRI was not readily available and could have delayed urgent treatment. Furthermore, multiple case reports and series from resource-limited settings have successfully relied on clinical diagnosis, serology, and treatment response in scrub typhus myocarditis without adverse outcomes [20]. Therefore, a clinical diagnosis was sufficient, and omitting MRI or biopsy was appropriate and aligned with established practice.

## Conclusions

This case underscores that scrub typhus, though often a mild febrile illness, can present with serious yet reversible cardiac complications such as myocarditis and complete heart block. In this patient, hypothyroidism obscured the diagnosis and delayed suspicion of an infectious cause. Scrub typhus-induced myocarditis was presumed based on clinical presentation and supported by echocardiographic findings. The complete atrioventricular block resolved rapidly following doxycycline therapy, highlighting its reversible nature and distinguishing it from more persistent causes. Clinicians in endemic areas should maintain a high index of suspicion for scrub typhus in patients with febrile bradyarrhythmias and incorporate it into local diagnostic algorithms to avoid unnecessary interventions and missed diagnoses. Follow-up with periodic clinical evaluation and ECG (with or without echocardiography) for at least three to six months is advisable to detect recurrence or late-onset sequelae. Finally, given the unpredictability of severe cardiac involvement, incorporating routine cardiac screening (e.g., ECG) into febrile illness evaluations in endemic settings could facilitate early detection and improve outcomes, particularly in resource-limited regions.
